# Detecting Metachanges in Data Streams from the Viewpoint of the MDL Principle

**DOI:** 10.3390/e21121134

**Published:** 2019-11-20

**Authors:** Shintaro Fukushima, Kenji Yamanishi

**Affiliations:** Department of Mathematical Informatics, Graduate School of Information Science and Technology, The University of Tokyo, 7-3-1 Hongo, Bunkyo-ku 113-8656, Japan; yamanishi@mist.i.u-tokyo.ac.jp

**Keywords:** change detection, change of change, data stream, minimum description length principle, code length

## Abstract

This paper addresses the issue of how we can detect changes of changes, which we call *metachanges*, in data streams. A metachange refers to a change in patterns of when and how changes occur, referred to as “metachanges along time” and “metachanges along state”, respectively. Metachanges along time mean that the intervals between change points significantly vary, whereas metachanges along state mean that the magnitude of changes varies. It is practically important to detect metachanges because they may be early warning signals of important events. This paper introduces a novel notion of metachange statistics as a measure of the degree of a metachange. The key idea is to integrate metachanges along both time and state in terms of “code length” according to the minimum description length (MDL) principle. We develop an online metachange detection algorithm (MCD) based on the statistics to apply it to a data stream. With synthetic datasets, we demonstrated that MCD detects metachanges earlier and more accurately than existing methods. With real datasets, we demonstrated that MCD can lead to the discovery of important events that might be overlooked by conventional change detection methods.

## 1. Introduction

### 1.1. Purpose of This Paper

In this study, we are concerned with detecting changes in data streams. The goal of *change detection* is to detect the time points at which the nature of the data-generating mechanism significantly changes.

Thus far, many algorithms have been proposed to detect change points in data streams (e.g., [1,2,3,4,5,6,7,8,9,10,11]), and several studies addressed or have been related to the issue of changes of changes [12,13,14,15,16,17,18]. In this paper, we refer to the changes of changes as *metachanges*. A metachange refers to a change in the pattern of when or how changes occur. It is practically important to detect metachanges because they may be early warning signals of important events [12,13]. Metachanges have been treated from a viewpoint of *metachanges along time*. Metachanges along time indicate that the interval significantly varies between the change points. Such metachanges were called *burstiness* [12] and *volatility* [13] in previous studies. The detection of metachanges along time provides users with useful information from data streams. For example, in a machine in a manufacturing factory, a decrease in the interval between change points might be a sign of a serious failure.

There is also another type of metachange: *metachanges along state*. Here, “state” refers to the parameter value of the probability density function of a distribution. We consider a situation where change points $t_{1},\ldots$ are detected for a data stream $y_{1},y_{2},\ldots$, and $y_{t}$ is drawn from $p_{y}\left(y_{t};\eta \right)$. Here, $p_{y}$ is a probability density function of distributions, and η is the associated parameter. Note that η is called *state* in this paper, and it varies before and after a change point. A metachange along state means a change of how significantly η varies before and after a change point. Metachanges along state might provide information such as changes of magnitude and velocity, which indicate an important change in the underlying data-generating mechanism. For example, in a machine in a manufacturing factory, a shift to an abrupt (sudden) change from a gradual (incremental) change [19], or its inverse shift, might be a sign of serious events.

**Figure 1:**
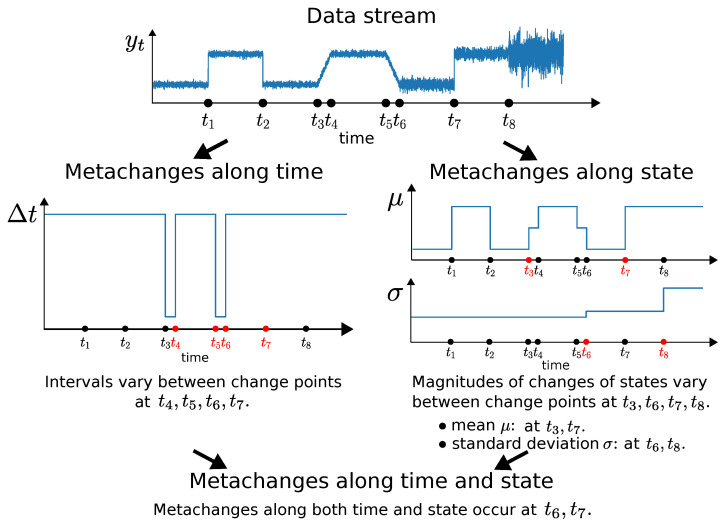
Conceptual illustration of metachanges.

A conceptual illustration of metachanges is shown in Figure 1, where the upper graph shows a data stream $y_{1},\ldots$ and change points ${\left\{t_{i}\right\}}_{i=1}^{8}$ on the horizontal axis. The lower left graph shows intervals between change points $\Delta t=t_{i}-t_{i-1}$ on the vertical axis. Metachanges along time occur at $t_{4},t_{5},t_{6},t_{7}$: for example, $t_{4}-t_{3}$ is different from $t_{3}-t_{2}$ and $t_{2}-t_{1}$. The lower right graph shows the states estimated piecewisely between the change points. Here, we assume $y_{t}$ is drawn from the univariate normal distribution $p_{y}\left(y_{t};\mu ,\sigma \right)$, where μ is the mean and σ is the standard deviation. In this case, (μ,σ) is a state. In Figure 1, because there is no significant change in the magnitude of state change between $t_{1}$ and $t_{2}$, a metachange along state does not occur at $t_{2}$. However, there is a significant change in the magnitude of change of μ between $t_{2}$ and $t_{3}$: thus, a metachange along state occurs at $t_{3}$. Because the magnitudes of the changes of μ and σ are almost the same between $t_{3}$ and $t_{4}$, a metachange along state does not occur at $t_{4}$. Using the same procedure, we conclude that metachanges along state occur at $t_{3}$ and $t_{7}$ with respect to μ. Moreover, metachanges along state occur at $t_{6}$ and $t_{8}$ with respect to σ: the magnitude of the change of standard deviations around $t_{6}$ ($t_{8}$) is greater than those around $t_{5}$ ($t_{7}$). As a result, metachanges along state occur at $t_{3},t_{6},t_{7},$ and $t_{8}$. We can infer that metachanges along both time and state occur at $t_{6}$ and $t_{8}$, by combining the metachanges along time and state.

Metachanges along time have been investigated in previous studies [12,13], and, although there have been several studies related to metachanges along state [14,15,16,17,18], the focus of these studies was not on metachanges along state in particular. The purpose of this paper is to propose a framework and an approach to detect metachanges along time and state from a unified view with the minimum description length (MDL) [20]. Therefore, our framework and approach not only include previous notions such as *burstiness* [12] and *volatility* [13] but also extend these notions to metachanges along state. MDL asserts that the best statistical decision strategy is the one that compresses the data best. Description and coding with MDL are suitable for quantifying changes, and they enable us to easily integrate the code lengths of time and state.

### 1.2. Related Work

Change detection has been extensively explored in the area of data mining. Thus far, several methods have been proposed to detect metachanges in data streams [12,13], and there have been several studies related to metachanges along state [14,15,16,17,18].

Kleinberg [12] and Huang et al. [13] proposed algorithms for detecting metachanges along time. Kleinberg [12] proposed an algorithm to detect bursts in a time series. This algorithm assumes that intervals between successive events are drawn from an exponential distribution. The discretized values of the parameters of the exponential distribution are regarded as states. For intervals between successive events, states are estimated with dynamic programming. Changes of state indicate changes of intervals between the successive events. Huang et al. [13] proposed an algorithm, called the *volatility detector*, which detects changes of rates of change. The volatility detector prepares two buckets, called the *buffer* and the *reservoir*, to store intervals between change points. The intervals are put into the buffer sequentially. When the buffer is full, an interval is dropped from the buffer and moved to the reservoir in a first-in-first-out fashion. The reservoir stores the dropped interval by randomly replacing one of its stored intervals. If the ratio of variances of the buffer and the reservoir is over or under the specified threshold, the algorithm judges that the intervals change between change points. The authors called this event *volatility shift*. Both the burst detector and volatility detector are assumed to be used in two steps. That is, change points are detected with other change detection algorithms, and then changes of intervals between the change points are detected. While the burst detector works in an offline fashion, the volatility detector works in an online fashion.

Moreover, there have been several studies related to metachanges along state [14,15,16,17,18]. Aggarwal [15] introduced *velocity density estimation* to understand, visualize, and determine trends in the evolution of fast data streams. Spiliopoulou et al. [16,17] proposed an algorithm, called MONIC, to model and track cluster transitions. Ntoutsi et al. [18] proposed an algorithm, called FINGERPRINT, to summarize cluster evolution. Huang et al. [14] proposed a change type detector, intended to categorize change types into three relative types, some of which correspond to concept drifts proposed in [19]. Although their algorithms [14,15,16,17,18] are related to metachanges along state, they are not intended to characterize and detect metachanges directly. In addition, many change detection algorithms have been proposed based on detecting changes of state (e.g., [6,7,8,9,21,22]). The dynamic model selection [6,7] is the seminal work to apply MDL to the task of dynamic model selection and change detection. The MDL change statistics [8], SCAW [9], and STREAMKRIMP [22] are change detection algorithms with MDL. However, these algorithms are not intended to characterize and detect metachanges along state directly.

### 1.3. Significance of This Paper

In the context of Section 1.1 and Section 1.2, the contributions of this paper are summarized in the following subsections.

#### 1.3.1. Proposal of Concept of Metachange

To detect changes of changes in data streams, we define a concept of *metachanges* along both time and state. Previous studies [12,13] considered metachanges along time only. In this paper, we deal with metachanges along both time and state. Metachanges along time include the notions proposed in previous studies such as burstiness [12] and volatility [13]. Metachange along state could capture changes of changes of the parameters of distribution between change points.

Our concept of *metachange* can detect the potential change of changes in data streams, which was overlooked by previous studies.

#### 1.3.2. Novel Algorithm for Detection of Metachanges

We define *metachange statistics* along both time and state. There is a challenge to combining the metachange statistics along time and those along state. In this paper, these statistics are defined based on the MDL principle. Metachange statistics along time (MCAT) is defined as the code length of an interval between the change points, whereas metachange statistics along state (MCAS) is defined as the difference between the predictive code length and the normalized maximum likelihood (NML) code length [23] after a change. It is possible to simply add these statistics because they are defined as code lengths, which enables us to detect metachanges along both time and state in a unified manner.

## 2. Theoretical Background of Metachange Statistics

In this section, we consider how to encode both intervals between change points and states around the change points. We assume that for a data stream $y_{1},\;y_{2},\;\ldots$ change points $t_{1},\;\ldots$ are detected and that the intervals between change points $x_{i}=t_{i}-t_{i-1}$ and $y_{t}$ are drawn, respectively, from

$$x_{i}∼p_{x}\left(x_{i};\xi \right),\;y_{t}∼p_{y}\left(y_{t};\;\eta \right),$$

where $p_{x}$ and $p_{y}$ are probability density functions of distributions and ξ and η are the associated parameters. Finally, η is the *state* whose metachanges are addressed in this paper.

### 2.1. Definitions of Metachanges

In this subsection, we give definitions of metachanges.

**Definition** **1.** *(Metachange along time) For intervals between change points $x_{1},\;x_{2},\;\ldots$, we say that a metachange along time occurs at a change point $t_{i}$ for a threshold parameter $\delta_{t}>0$ if and only if*

(1) $$\begin{array}{c} q_{1}\to q_{2}\;\;\mathrm{at}\;\;t=t_{i},\\ d\left(q_{1},q_{2}\right)>\delta_{t},\;q_{1},q_{2}\in F_{t},\;F_{t}=\left\{p_{x}\left(x;\xi \right)\right\}, \end{array}$$

*where $q_{1}$ and $q_{2}$ are distributions of intervals. $q_{1}\to q_{2}$ means that $x_{t}∼q_{1}$ at $t=t_{i-1}$ and $x_{t}∼q_{2}$ at $t=t_{i}$. d is a distance function between the probability density functions.*

**Definition** **2.** *(Metachange along state) For a data stream $y_{1},y_{2},\ldots$, we say that a* metachange along state *occurs at a change point $t_{i}$ for a threshold parameter $\delta_{s}>0$ if and only if*

(2) $$\begin{array}{c} q_{1}\to q_{2}\;\;\mathrm{at}\;\;t=t_{i-1},\;q_{2}\to q_{3}\;\;\mathrm{at}\;\;t=t_{i},\\ |d\left(q_{2},q_{3}\right)-d\left(q_{1},q_{2}\right)|>\delta_{s},\;q_{1},q_{2},q_{3}\in F_{s},\;F_{s}=\left\{p_{y}\left(y;\eta \right)\right\}, \end{array}$$

*where $q_{1}$, $q_{2}$, and $q_{3}$ are distributions of values of the data stream. Equation *(2)* means that $y_{t}∼q_{1}$ at $t=t_{i-2},\ldots ,t_{i-1}-1$, $y_{t}∼q_{2}$ at $t=t_{i-1},\ldots ,t_{i}-1$, and $y_{t}∼q_{3}$ at $t=t_{i}\ldots ,t_{i+1}-1$. Here, d is the same as that in Definition 1.*

**Definition** **3.** *(Integrated metachange) For a change point $t_{i}$, we say that an* integrated metachange *occurs at $t_{i}$ if and only if Equation *(1)* or Equation *(2)* holds.*

### 2.2. Problem Setting

In this subsection, we consider a situation where (m+1) change points $t_{1},\ldots ,t_{m+1}$ are given. We consider how to encode $x_{i}$ and $y_{t}$ as shortly as possible. The ideal code length required for encoding $x_{i}$ is given by what we call the predictive code length, which is the sum of the negative logarithm of its predictive density $p_{x}$ at each time point, defined as follows:

(3) $$\min_{{\left\{{\hat{\xi }}_{x^{i-1}}\right\}}_{i=1}^{m}}\sum_{i=1}^{m}\;-\log p_{x}\left(x_{i};{\hat{\xi }}_{x^{i-1}}\right),$$

where ${\hat{\xi }}_{x^{i-1}}$ are estimated at each change point. Similarly, the ideal code length required for encoding $y_{t}$ around change points is given by the predictive code length as follows:

(4) $$\min_{{\left\{{\hat{\eta }}_{y^{t-1}}|t\in \mathrm{neighbor}\left(t_{i}\right)\right\}}_{i=1}^{m}}\sum_{i=1}^{m}\sum_{t\in \mathrm{Neighbor}(t_{i})}\;-\log p_{y}\left(y_{t};{\hat{\eta }}_{y^{t-1}}\right),$$

where $\mathrm{Neighbor}(t_{i})$ indicates the neighborhood of a change point $t_{i}$. In practice, as explained in Section 3.3, $\mathrm{Neighbor}\left(t_{i}\right)=\left[t_{i}-h,t_{i}+h\right],h\in N$. ${\hat{\xi }}_{x^{i-1}}$ and ${\hat{\eta }}_{y^{t-1}}$ are estimated using $x^{i-1}=x_{1}\ldots x_{i-1}$ and $y^{t-1}=y_{1}\ldots y_{t-1}$, respectively. A change of ${\hat{\eta }}_{y^{t-1}}$ indicates a change of state. Detection of a metachange along time is asserted as a problem of detection of a change of ${\hat{\xi }}_{x^{i-1}}$ in Equation (3). On the other hand, detection of a metachange along state is asserted as a problem of detection of a change of how ${\hat{\eta }}_{y^{t-1}}$ in Equation (4) changes around a change point between change points.

## 3. Metachange Detection Algorithm

**Figure 2:**
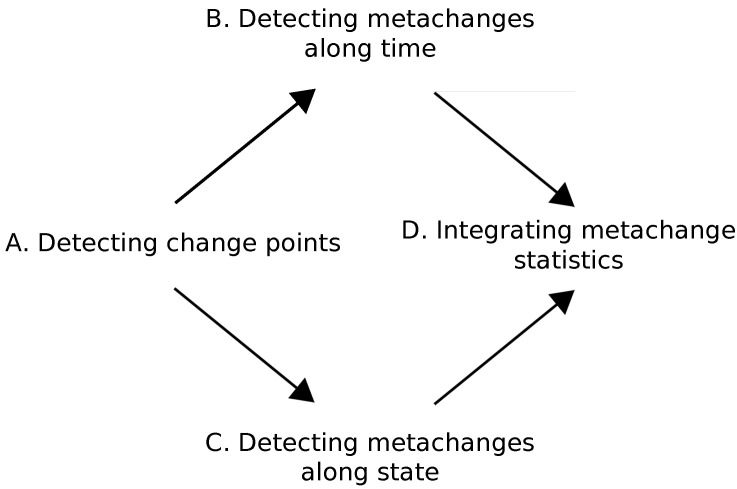
Schematic of the proposed metachange detection algorithm (MCD) algorithm.

In this section, we present our online algorithm called metachange detection algorithm (MCD) for detecting metachanges along both time and state. We consider how to achieve Equations (3) and (4) in an online fashion. A schematic description of MCD is shown in Figure 2.

First, we detect change points from data stream (A). Next, we concurrently detect metachanges along time (B) and along state (C). We introduce metachange statistics to quantify these metachanges. Finally, we integrate the metachange statistics along time and state into a statistics (D).

The key challenge of detecting metachanges along time and state is how to describe and integrate them. Our approach describes both metachanges as code lengths with MDL; therefore, it is easy to combine them.

### 3.1. Detecting Change Points

First, we detect change points $t_{1},\;t_{2},\;\ldots$. As our proposed algorithm MCD works in an online fashion, it is necessary for the change detection algorithm to work in an online fashion (e.g., [1,2,3,4,8,9]). In general, MCD is prone to errors by the change detection algorithm and its threshold parameter. We empirically investigate and discuss this point in detail in Section 4.

### 3.2. Detecting Metachanges along Time

For the detected change points $t_{1}\;\ldots$, let us consider intervals between the successive change points $I_{i}=\left[t_{i-1},\;t_{i}-1\right]$, with length $x_{i}=t_{i}-t_{i-1}$. For an interval sequence $x^{i}=x_{1}\ldots x_{i}$, we consider how to achieve Equation (3) in an online fashion. We define metachange along time (MCAT) $a_{t_{i}}$ as the predictive code length

(5) $$a_{t_{i}}\overset{\mathrm{def}}{=}-\log p_{x}\left(x_{i};{\hat{\xi }}_{x^{i-1}}\right),$$

where $p_{x}\in F_{t}$, $F_{t}=\left\{p_{x}\left(x;\xi \right)\right\}$ is a parametric class of probability distribution, and ${\hat{\xi }}_{x^{i-1}}$ is estimated using $x^{i-1}=x_{1}\ldots x_{i-1}$. For example, we can estimate ${\hat{\xi }}_{x^{i-1}}$ as the maximum likelihood estimator. To deal with nonstationary data streams, we use the online discounting maximum likelihood estimator [24]

(6) $$\begin{array}{c} {\hat{\xi }}_{x^{i-1}}=\underset{\xi }{\mathrm{argmax}}\;\sum_{t=1}^{i-1}\;r{\left(1-r\right)}^{i-1-t}\log p_{x}\left(x_{t};\xi \right), \end{array}$$

where 0<r<1 is a discounting parameter. An increase in *r* has a greater effect on forgetting past data.

In this paper, we introduce a parametric class of the exponential distribution

(7) $$F_{t}=\left\{p_{x}\left(x;\xi \right)=\xi \exp\left(-\xi x\right),\;\xi >0\right\}.$$

By substituting Equation (7) into Equation (6), we get

(8) $$\begin{array}{cc} {\hat{\xi }}_{x^{i-1}} & =\underset{\xi }{\mathrm{argmax}}\sum_{t=1}^{i-1}r{\left(1-r\right)}^{i-1-t}\log\left(\xi \exp\left(-\xi x_{t}\right)\right)\\ & =\underset{\xi }{\mathrm{argmax}}\sum_{t=1}^{i-1}r{\left(1-r\right)}^{i-1-t}\left(\log\xi -\xi x_{t}\right). \end{array}$$

The inside of argmax in the right-hand side of Equation (8) is expanded as

(9) $$\begin{array}{cc} \sum_{t=1}^{i-1}r{\left(1-r\right)}^{i-1-t}\left(\log\xi -\xi x_{t}\right) & =r\log\xi \sum_{t=1}^{i-1}{\left(1-r\right)}^{i-1-t}-r\xi \sum_{t=1}^{i-1}{\left(1-r\right)}^{i-1-t}x_{t}\\ \; & =r\log\xi \frac{1-{\left(1-r\right)}^{i-1}}{r}-r\xi \sum_{t=1}^{i-1}{\left(1-r\right)}^{i-1-t}x_{t}\\ \; & =\log\xi \left(1-{\left(1-r\right)}^{i-1}\right)-r\xi \sum_{t=1}^{i-1}{\left(1-r\right)}^{i-1-t}x_{t}. \end{array}$$

The right-hand side of Equation (9) is maximized by deriving it with respect to ξ. As a result, we obtain the following optimal solution:

(10) $${\hat{\xi }}_{x^{i-1}}=\frac{1-{\left(1-r\right)}^{i-1}}{r\sum_{t=1}^{i-1}{\left(1-r\right)}^{i-1-t}x_{t}}.$$

Thus, by substituting Equation (10) into Equation (5), MCAT at $t_{i}$ is

(11) $$\begin{array}{cc} a_{t_{i}} & =-\log p_{x}\left(x_{i};{\hat{\xi }}_{x^{i-1}}\right)=-\log{\hat{\xi }}_{x^{i-1}}+{\hat{\xi }}_{x^{i-1}}x_{i}. \end{array}$$

In practice, we judge that a metachange occurs along time when MCAT changes greatly between the change points. Technically, we use the change rate of MCAT: a metachange occurs along time when $|\left(a_{t_{i}}-a_{t_{i-1}}\right)/a_{t_{i-1}}|>\epsilon_{t}$ holds, where $\epsilon_{t}>0$ is a threshold parameter. We call the algorithm described above as the *metachange detection along time algorithm* (MCD-T).

As for computational cost of MCAT, Equation (10) is written as

$${\hat{\xi }}_{x^{i-1}}=\frac{1-{\left(1-r\right)}^{i-1}}{r\;s_{i-1}},$$

where

$$s_{i-1}\overset{\mathrm{def}}{=}\sum_{j=1}^{i-1}{\left(1-r\right)}^{i-1-j}x_{j}.$$

$s_{i}$ and $s_{i-1}$ satisfy the following relation:

$$s_{i}=\left(1-r\right)s_{i-1}+x_{i}.$$

Therefore, the computational cost of MCAT $a_{t_{i}}$ is O(i).

**Figure 3:**
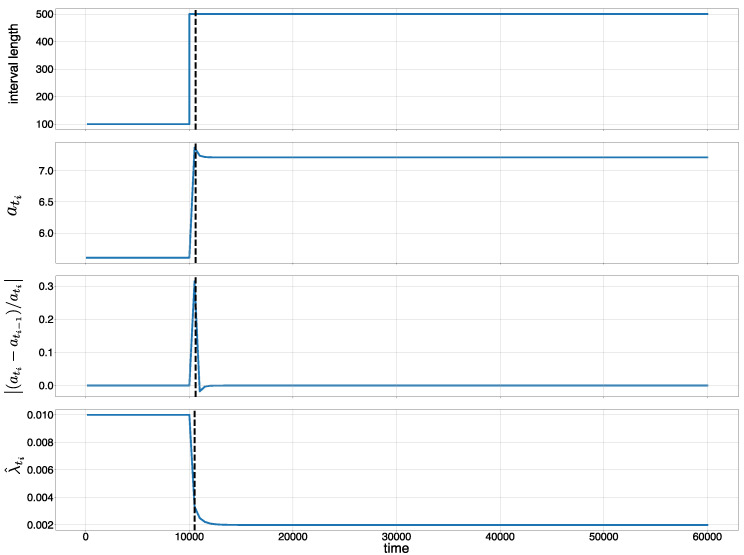
Metachange statistics along time (MCAT): (**top**) time interval at each change point; (**second**) MCAT $a_{t_{i}}$; (**third**) change rate of MCAT $|\left(a_{t_{i}}-a_{t_{i-1}}\right)/a_{t_{i-1}}|$; and (**bottom**) the estimated parameter of the exponential distribution $\hat{\lambda }$. The discounting parameter r=0.5.

Example:

We consider a data stream with a length of 200 time intervals between change points: $x_{i}=100\;\left(i=1,\;\ldots ,\;100\right)$ and $x_{i}=500\;\left(i=101,\;\ldots ,\;200\right)$. This means that there are 201 change points ${\left\{t_{i}\right\}}_{i=1}^{201}$. If we assume $t_{1}=100$, then $t_{2}=200,\;\ldots ,\;t_{101}=$ 10,100, $t_{102}=$ 10,600, $t_{103}=$ 11,100, …, $t_{201}=$ 60,100. Then, $x_{i}$ is calculated as $x_{i}=t_{i}-t_{i-1}$. Figure 3 shows the time intervals at the change points (Figure 3, top), MCATs $a_{t_{i}}$ (Figure 3, second graph), the change rate of MCATs $|\left(a_{t_{i}}-a_{t_{i-1}}\right)/a_{t_{i-1}}|$ (Figure 3, third graph), and ${\hat{\xi }}_{x^{i-1}}$ (Figure 3, bottom). We observe in Figure 3 that we can detect the metachange along time when we choose a suitable threshold $\epsilon_{t}$. Here, the discounting parameter is set to r=0.5.

### 3.3. Detecting Metachanges Along State

For a change point $t_{i}$ detected in Section 3.1, we consider how to achieve Equation (4) in an online fashion. We consider a subset of time around $t_{i}$ for $\mathrm{Neighbor}(t_{i})$ in Equation (4). The subset is denoted by $J_{i}=\left[t_{i}-h,\;t_{i}+h\right]$, where h∈N is a window size. Thus, we consider a sequence $y_{t_{i}-h}^{t_{i}+h}=y_{t_{i}-h}\;\ldots \;y_{t_{i}+h}$, with length n=2h+1. We introduce a parametric class of probability distributions $F_{s}=\left\{p_{y}\left(Y;\eta \right);\eta \in H\right\}$. Here, *Y* is a random variable and η is a real-valued parameter. *H* is the associated parameter space.

Next, we define metachange statistics along state (MCAS) at change point $t_{i}$. First, two statistics, $b_{t_{i}}^{+}$ and $b_{t_{i}}^{-}$, are introduced. These are defined as the difference between two code lengths for $y_{t_{i}+1}^{t_{i}+h}$: one is the “expected” code length, estimated using the parameter change at $t_{i-1}$ and the estimated parameter with $y_{t_{i}-h}^{t_{i}-1}$. The other is the code length with the parameter estimated in terms of $y_{t_{i}+1}^{t_{i}+h}$. Formally, $b_{t_{i}}^{\pm }$ is defined as the difference between the predictive code length and the NML code length [20] after the change point. The former is calculated as the predictive code length, which is the total code length for encoding $y_{t_{i}+1}^{t_{i}+h}$ in a predictive way, using the estimated parameter $\eta^{\pm }$ as follows:

(12) $$\frac{1}{h}\sum_{t=t_{i}+1}^{t_{i}+h}-\log p_{y}\left(y_{t};{\hat{\eta }}^{\pm }\right),$$

where ${\hat{\eta }}^{\pm }$ is defined as

(13) $${\hat{\eta }}^{\pm }\overset{\mathrm{def}}{=}{\hat{\eta }}_{y_{t_{i}-h}^{t_{i}-1}}\pm \left({\hat{\eta }}_{y_{t_{i-1}+1}^{t_{i-1}+h}}-{\hat{\eta }}_{y_{t_{i-1}-h}^{t_{i-1}-1}}\right),$$

which indicates the parameter change to *the same side* and *the opposite side* in the same way as the previous change point $t_{i-1}$. Here, ${\hat{\eta }}_{y_{\tau_{1}}^{\tau_{2}}}$ means the maximum likelihood estimator of η using $y_{\tau_{1}}^{\tau_{2}}=y_{\tau_{1}}\ldots y_{\tau_{2}}$.

The latter is calculated as the NML code length, which is defined as the negative logarithm of the NML distribution [20]:

(14) $$\frac{1}{h}\left(\sum_{t=t_{i}+1}^{t_{i}+h}-\log p_{y}\left(y_{t};{\hat{\eta }}_{y_{t_{i}+1}^{t_{i}+h}}\right)+\log C_{h}\right).$$

The difference between Equation (12) and Equation (14) is given by

(15) $$\begin{array}{c} b_{t_{i}}^{\pm }\overset{\mathrm{def}}{=}\frac{1}{h}\left\{\sum_{t=t_{i}+1}^{t_{i}+h}\left(-\log p_{y}\left(y_{t};{\hat{\eta }}^{\pm }\right)+\log p_{y}\left(y_{t};{\hat{\eta }}_{y_{t_{i}+1}^{t_{i}+h}}\right)\right)-\log C_{h}\right\}, \end{array}$$

where $C_{h}=\sum_{z_{t_{i}+1}^{t_{i}+h}}\max_{\eta }p_{y}\left(z_{t_{i}+1}^{t_{i}+h};\eta \right)$ in Equation (15) is computed using Rissanen’s approximation formula under some regularity conditions [23]:

$$\log C_{h}\approx \frac{k}{2}\log\frac{h}{2\pi }+\log\int \sqrt{\left|I(\theta )\right|}\;d\theta ,$$

where *k* is the dimension of *H* and $I\left(\theta \right)\overset{\mathrm{def}}{=}E_{\eta }\left[-\partial^{2}\log p_{y}\left(Y;\eta \right)/\partial \eta_{i}\partial \eta_{j}\right]$ is the Fisher information matrix at the parameter value η. Intuitively, Equation (15) quantifies the redundant code length for coding $y_{t_{i}+1}^{t_{i}+h}$ with the parameters estimated in terms of the parameter change at $t_{i-1}$ and the parameter values in the former part of $t_{i}$.

Finally, we define MCAS as

(16) $$b_{t_{i}}\overset{\mathrm{def}}{=}\min\left(b_{t_{i}}^{+},b_{t_{i}}^{-}\right),$$

which means that metachanges along state are quantified by the relative magnitude of changes in the parameters in this paper. The computational cost of MCAS is O(h)=O(1). We judge that a metachange along state occurs at $t_{i}$ when $b_{t_{i}}>\epsilon_{s}$ holds, where $\epsilon_{s}>0$ is a threshold parameter. We call the algorithm described above as the *metachange detection along state algorithm* (MCD-S).

Example:

We generate a data stream with length 11,250:

$$y_{t}∼\left\{\begin{array}{c} N(0.0,\;0.05)\;(t=1,\;\ldots ,\;1000)\\ N(1.0,\;0.05)\;(t=1001,\;\ldots ,\;2000)\\ N(0.0,\;0.05)\;(t=2001,\;\ldots ,\;3000)\\ N(1.0,\;0.05)\;(t=3001,\;\ldots ,\;4000)\\ N((t-4001)/1000,\;0.05)\;(t=4001,\;\ldots ,\;5000)\\ N(0.0,\;0.05)\;(t=5001,\;\ldots ,\;6000)\\ N((t-6000)/1000,\;0.05)\;(t=6001,\;\ldots ,\;7000)\\ N(1.0,\;0.05)\;(t=7001,\;\ldots ,\;8000)\\ N(1-(t-8000)/250,\;0.05)\;(t=8001,\;\ldots ,\;8250)\\ N(0.0,\;0.1)\;(t=8251,\;\ldots ,\;9250)\\ N(1.0,\;0.1)\;(t=9251,\;\ldots ,\;10,250)\\ N(1.0,\;0.3)\;(t=10,251,\;\ldots ,\;11,250) \end{array}\right.,$$

where N(μ,σ) denotes the probability density function of the univariate normal distribution with mean μ and standard deviation σ.

**Figure 4:**
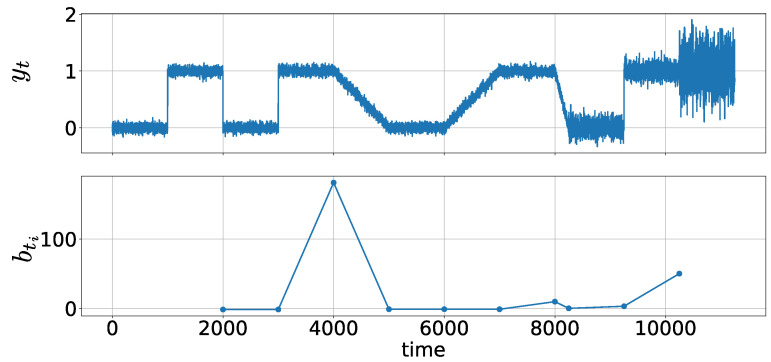
Metachange statistics along state (MCAS): (**top**) data stream $y_{t}$; and (**bottom**) MCAS $b_{t_{i}}$. Window size h=200.

Figure 4 shows data stream $\left\{y_{t}\right\}$ (Figure 4, top) and statistics $\left\{b_{t_{i}}\right\}$ (Figure 4, bottom). The parameter is set to h=200. True change points occur at 1001, 2001, 3001, 4001, 5101, 6001, 7001, 8001, 8251, 9251, and 10,251. Figure 4 shows that the statistics $b_{t_{i}}$ increase when there is a change in how parameters behave around a change point between successive change points. At $t_{2}=2001$ and $t_{3}=3001$, $b_{t_{i}}$ are relatively small, which shows that parameter changes (i.e., their magnitudes) do not differ much between $t_{1}=1001$ and $t_{2}=2001$ and between $t_{2}=2001$ and $t_{3}=3001$. However, $b_{t_{i}}$ increases at $t_{4}=4001$ because the change shifts to a gradual change from an abrupt one. These results indicate that MCAS provides information regarding changes in the behavior around the change points.

### 3.4. Integrating Metachange Statistics

Finally, we consider how to integrate MCAT $a_{t_{i}}$ and MCAS $b_{t_{i}}$ at a change point $t_{i}$. Because $a_{t_{i}}$ and $b_{t_{i}}$ are code lengths, they can be summed. Therefore, we propose adding $a_{t_{i}}$ and $b_{t_{i}}$ with weighting. Integrated metachange (MCI) $s_{t_{i}}$ at $t_{i}$ is defined as

(17) $$s_{t_{i}}\overset{\mathrm{def}}{=}a_{t_{i}}+\lambda b_{t_{i}},$$

where λ∈R is a hyperparameter. We should carefully choose λ with data. In Section 4.3, λ is determined using a grid search.

In practice, we judge that a metachange along both time and state occur at $t_{i}$ when MCI greatly changes between the change points. As in the case of metachanges along time in Section 3.2, we use the change rate of MCI: a metachange along both time and state occurs at $t_{i}$ if $|\left(s_{t_{i}}-s_{t_{i-1}}\right)/s_{t_{i-1}}|>\epsilon_{\mathrm{ts}}$, where $\epsilon_{\mathrm{ts}}>0$ is a threshold parameter.

We call the overall algorithm described above *MCD*; it is summarized in Algorithm 1.

**Algorithm 1** MCD.**Input:***r*: discounting parameter (0<r<1), *h*: window size, $\epsilon_{\mathrm{ts}}$: threshold parameter **Output:**$a_{t_{i}}$: metachange statistics along time, $b_{t_{i}}$: metachange statistics along state, $s_{t_{i}}$: integrated metachange statistics. 1:i=12:**for**t=1,…**do**3:   Input $y_{t}$.4:   Detect change point with a change detection algorithm.5:   **if** *t* is change point **then**6:        $t_{i}\leftarrow t$.7:        $x_{i}\leftarrow t_{i}-t_{i-1}$.8:        Calculate metachange statistics along time $a_{t_{i}}$ according to Equation (11).9:        Calculate metachange statistics along state $b_{t_{i}}$ according to Equation (16).10:     Calculate integrated metachange statistics $s_{t_{i}}$ according to Equation (17).11:      Raise an alarm if and only if $|\left(s_{t_{i}}-s_{t_{i-1}}\right)/s_{t_{i-1}}|>\epsilon_{\mathrm{ts}}$.12:      i←i+1.13:   **end if**14:**end for**

## 4. Experiment

We conducted five experiments to confirm the effectiveness of the proposed algorithm MCD (https://github.com/s-fuku/metachange).

### 4.1. Synthetic Dataset 1 (Metachanges along Time)

We defined six levels of time intervals between change points referring to the work in [13,25]. The interval lengths were 100,000, 50,000, 10,000, 5000, 1000, and 500. The change points were set using a Bernoulli distribution oscillating between μ=0.2 and μ=0.8. For each combination of two intervals, we generated the streams based on the scheme above. Each stream contained 100 change points. In what follows, $L_{1}$ and $L_{2}$ indicate the first and second interval lengths, respectively.

**Figure 5:**
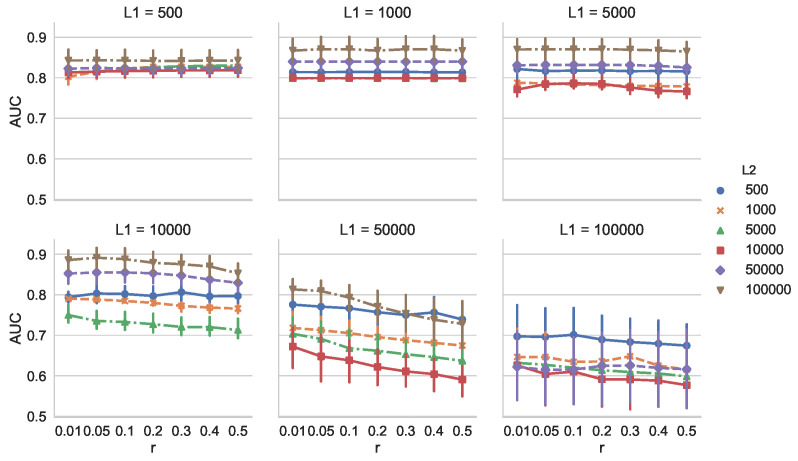
Dependency of AUC on discounting parameter *r* for MCD-T on Synthetic Dataset 1.

**Figure 6:**
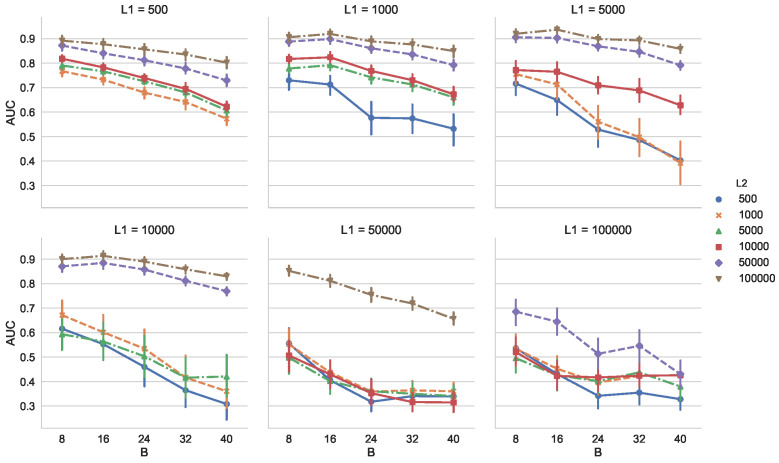
Dependency of AUC on the buffer size *B* (= the reservoir size *R*) for VD on Synthetic Dataset 1.

We confirmed the effectiveness of MCD by comparing it with a volatility detector (VD) [13]. We used the SEED algorithm [13] and the sequential MDL-change statistics algorithm (SMDL) [8] for change detection. SEED was based on ADWIN2 [21] and its parameters were set to $\delta =0.05,\;\Gamma =75,\;\hat{\epsilon }=0.025$, and α=0.025, which are the same as those in [13]. The window size *w* of SMDL was set to $w=0.2L_{1}$, and the threshold parameter ϵ was set to ϵ=0.01. For the Bernoulli distribution, the change score $\Psi_{t}$ of SMDL at time *t* was calculated as

$$\begin{array}{cc} \Psi_{t} & =-{\hat{\mu }}_{0}\log{\hat{\mu }}_{0}-\left(1-{\hat{\mu }}_{0}\right)\log\left(1-{\hat{\mu }}_{0}\right)\\ \; & \;-\frac{1}{2}\left(-{\hat{\mu }}_{1}\log{\hat{\mu }}_{1}-\left(1-{\hat{\mu }}_{1}\right)\log\left(1-{\hat{\mu }}_{1}\right)\right)-\frac{1}{2}\left(-{\hat{\mu }}_{2}\log{\hat{\mu }}_{2}-\left(1-{\hat{\mu }}_{2}\right)\log\left(1-{\hat{\mu }}_{2}\right)\right), \end{array}$$

where ${\hat{\mu }}_{0}=\sum_{i=t-w}^{t+w}y_{i}/\left(2w+1\right)$, ${\hat{\mu }}_{1}=\sum_{i=t-w}^{t-1}y_{i}/w$, and ${\hat{\mu }}_{2}=\sum_{i=t}^{t+w}y_{i}/\left(w+1\right)$. If $\Psi_{t}>\epsilon$, *t* is regarded as a change point. We determined that *t* was a change point if the change score $\Psi_{t}$ was the maximum. The parameter of MCD-T was set to r=0.2. Below, we discuss the dependency of MCD-T on *r* in Figure 5. For VD, buffer size B=32 and reservoir size R=32, which were the same as in [13]. We also discuss the dependency of VD on *B* and *R* below in Figure 6. In running SEED [13], we used the Java source code provided by the authors (https://www.cs.auckland.ac.nz/research/groups/kmg/DavidHuang.html). We started to use change points when its number reached B+R for MCD-T and VD because the buffer and the reservoir of VD are not full until B+R intervals arrive.

We investigated the trade-off between detection delay and accuracy in terms of benefit and false alarm rate, defined as in [8,26]. For MCD-T, we first fixed the threshold parameter $\epsilon_{t}$ and converted MCAT $\left\{a_{t_{i}}\right\}$ in Equation (11) to binary alarms $\left\{\alpha_{t_{i}}\right\}$. That is, $\alpha_{t_{i}}=𝟙\;(|\left(a_{t_{i}}-a_{t_{i-1}}\right)/a_{t_{i-1}}\left|>\epsilon_{t}\right)$, where 𝟙(t) denotes the binary function that takes 1 if and only if *t* is true. We evaluated MCD-T by varying $\epsilon_{t}$. We let τ be a maximum tolerant delay of metachange detection. When the metachange really started from $t^{*}$, we defined the *benefit* of an alarm at time *t* as

(18) $$b\left(t;t^{*}\right)=\left\{\begin{array}{cc} 1-\frac{|t-t^{*}|}{\tau } & (0\leq |t-t^{*}|<\tau ),\\ 0 & (\mathrm{otherwise}). \end{array}\right.$$

The number of *false alarms* was calculated as

(19) $$n\left(\alpha_{1}^{m}\right)\overset{\mathrm{def}}{=}\sum_{k=1}^{m}\alpha_{t_{k}}𝟙\left(b\left(t_{k},t^{*}\right)=0\right).$$

We visualized the performance by plotting the recall rate of the total benefit, *b*, against the false alarm rate, $n/\sup_{\epsilon_{t}}n$, with $\epsilon_{t}$ varying. Likewise, for VD, $\alpha_{t_{i}}$ was calculated using the *relative volatility* between the variances of the buffer and the reservoir by varying the threshold parameter β. We evaluated all four combinations of change detectors SEED and SMDL and metachange detectors MCD-T and VD by calculating the average and standard deviation of the area under the curve (AUC) of the benefit vs. FAR curves. The AUC scores were calculated over 50 sequences. The delay parameter was set to $\tau =5L_{2}$. Table 1 shows the average AUC scores. Table 1 shows that MCD-T with SEED or MCD-T with SMDL outperforms VD with SEED or VD with SMDL. This indicates the effectiveness of MCD-T.

Because MCD-T depends on discounting parameter *r* and the change detection algorithm used, we investigated these effects. First, we examined the dependency of AUC on *r* for all combinations of $L_{1}$ and $L_{2}$. We calculated AUC for 30 times with r=0.01,0.05,0.1,0.2,0.3,0.4, and 0.5. We used SEED [13] as the change detection algorithm, and its parameters were set to the same values as above. The dataset used was also the same as in the previous experiment. Figure 5 shows that, when $L_{1}$ is relatively small (e.g., $L_{1}=500,1000,5000,10,000$), AUC is not heavily dependent on *r*. When $L_{1}$ is larger, however, we observe that the larger *r* is, the smaller AUC is. This is because, with an increase of $L_{1}$, the number of false alarms of SEED also increases. In such situations, MCD-T is more prone to the false alarms when *r* is larger.

Figure 6 shows the dependency of AUC of VD on the buffer size *B* and the reservoir size *R* (B=R) for comparison. We calculated AUC for 50 times. We observe from Figure 6 that AUC decreases as *B* increases. In addition, we also see that MCD-T outperforms VD for various combinations of *r* and B(=R) by comparing Figure 5 with Figure 6.

**Figure 7:**
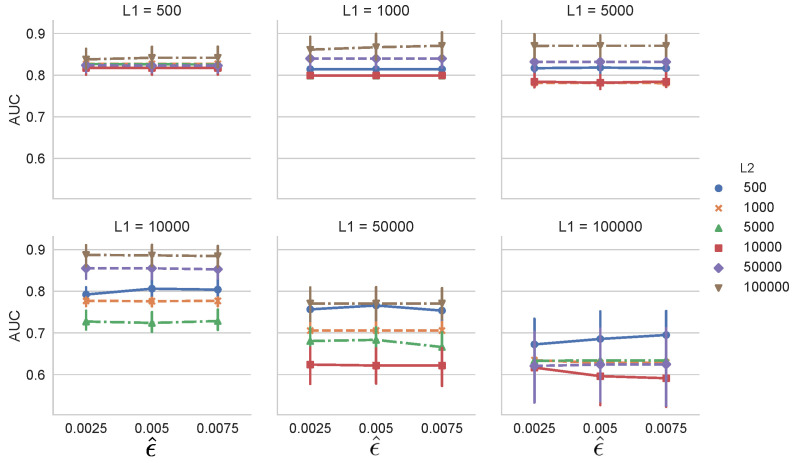
Dependency of AUC on threshold controlling parameter $\hat{\epsilon }$ of SEED [13] on Synthetic Dataset 1.

Next, we investigated the effect of the change detection algorithm used. We used SEED by changing the parameter $\hat{\epsilon }=0.0025,0.005$, and 0.0075. Other conditions and the dataset were the same as in the previous experiment. Here, $\hat{\epsilon }$ is a hyperparameter that controls the threshold parameter [13]. Figure 7 shows that AUC does not heavily depend on $\hat{\epsilon }$ for all combinations of $L_{1}$ and $L_{2}$. In general, the threshold parameter of the change detection algorithm controls the performance of MCD-T. Hence, it should be carefully set.

### 4.2. Synthetic Dataset 2 (Metachanges along State)

We generated a data stream with length 24L, where L=500,1000,2000. The generated data stream contained a metachange along state. In the former part, each datum was drawn from

(20) $$y_{t}∼\left\{\begin{array}{cc} N(0,\;0.1) & (t=1,\;\ldots ,\;L),\\ N(0.5,\;0.1) & (t=L+1,\;\ldots ,\;2L). \end{array}\right.$$

After we repeated the procedure 10 times, we obtained a subsequence with length 20L. In the latter part, each datum was drawn from

$$y_{t}∼\left\{\begin{array}{cc} N((t-20L)/2L,\;0.1)\; & (t=20L+1,\;\ldots ,\;21L),\\ N(0,0.1)\; & (t=21L+1,\;\ldots ,\;24L). \end{array}\right.$$

A metachange along state occurred at t=20L+1. For change detection, we employed four algorithms for comparison: (1) SMDL [8], a semi-instant method with the MDL change statistics; (2) ChangeFinder (CF) [1,2,4], a state-of-the-art method of abrupt change detection; (3) Bayesian online change point detection (BOCPD) [3], a retrospective online change point detection with a Bayesian scheme; and (4) ADWIN2 [21], adaptive windowing methods. As we assumed a situation where change and metachange mechanisms do not vary significantly, we decided to choose the best combinations of parameters of each change detection algorithm by grid search, as in [8,27]. We generated 10 sequences with the scheme above and calculated the F-scores for each combination of the following parameters:

- SMDL: Window size w=50,100 (L=500), w=100,200 (L=1000), w=200,400 (L=2000). Threshold parameter ϵ=0.1,0.2,0.3,0.4,0.5,0.6,0.7.
- CF: Discounting rate r=0.003,0.005,0.01,0.03,0.1. Threshold parameter δ=0,0.5,1.0,1.5,2.0 (regression orders $k_{1},k_{2}=3$, smoothing parameters $T_{1},T_{2}=5$).
- BOCPD: Parameter related to change intervals α=100,300,600. Threshold parameter ϵ=0.1,0.3.
- ADWIN2: Confidence parameter δ=0.1,0.2,0.3,0.4,0.5,0.6,0.7,0.8,0.9.

F-score is defined as the harmonic mean of *precision* and *recall*, which are calculated using the number of *true positives* (TP), *false positives* (FP), and *false negatives* (FN) as follows [9]: *TP* is the number of true change points that are τ-neighbors of estimated change points. Thus, *FP* and *FN* are calculated as FP=ℓ−TP and FN=m−TP, where *ℓ* and *m* are calculated as FP=ℓ−TP and FN=m−TP, where *ℓ* and *m* denotes the total number of estimated and true change points, respectively. Finally, we calculated recall=TP/(TP+FN) and precision=TP/(TP+FP) for each method. In this experiment, we set τ to 100.

After optimizing the parameters of each change detection algorithm, we generated 30 data streams with the scheme above and detected change points and the metachange. In the metachange detection, we compared MCD-S with SMDL. We chose SMDL for comparison because it calculates a change score at each time based on changes of parameters with MDL. Hence, a change rate of scores between change points is regarded as the degree of metachange along state. Hereafter, we refer to SMDL for metachange detection as SMDL metachange (SMDL-MC) and the window parameter as $w_{\mathrm{mc}}$. We calculated MCAS in Equation (16) for MCD-S and the change rate $|\left(\Psi_{t_{i}}-\Psi_{t_{i-1}}\right)/\Psi_{t_{i-1}}|$ for SMDL-MC. $\Psi_{t}$ is the *change score* at time *t* for a univariate normal distribution [8]:

$$\Psi_{t}=\frac{1}{2}\log\frac{{\hat{\sigma }}_{0}^{2}}{{\hat{\sigma }}_{1}{\hat{\sigma }}_{2}}+\log\frac{C_{2w_{\mathrm{mc}}}}{C_{w_{\mathrm{mc}}}^{2}},$$

where ${\hat{\sigma }}_{0}$, ${\hat{\sigma }}_{1}$, and ${\hat{\sigma }}_{2}$ are the maximum likelihood estimators of standard deviations calculated for $y_{t-w_{\mathrm{mc}}+1}^{t+w_{\mathrm{mc}}}$, $y_{t-w_{\mathrm{mc}}+1}^{t-1}$ and $y_{t}^{t+w_{\mathrm{mc}}}$, respectively. $C_{k}$ is the normalizer of the normalized maximum likelihood code length [20]

$$\log C_{k}=\frac{1}{2}\log\frac{16\mu_{\max}}{\pi \sigma_{\min}^{2}}+\frac{k}{2}\log\frac{k}{2e}-\log\Gamma \left(\frac{k-1}{2}\right),$$

where Γ is the gamma function. In this paper, $\mu_{\max}=2$ and $\sigma_{\min}=0.005$. The window parameters *h* of MCD-S and $w_{\mathrm{mc}}$ of SMDL-MC were set to $h,w_{\mathrm{mc}}=100$ (L=500), $h,w_{\mathrm{mc}}=200$ (L=1000), and $h,w_{\mathrm{mc}}=400$ (L=2000). In calculating the F-scores, the maximum tolerant delay was set to τ=0.5L.

Table 2 shows the average AUC values of MCD-S and SMDL-MC for the detection of metachanges along state at t=20L+1. The first and second rows in the header represent change detection and metachange detection algorithms, respectively. The best parameters for each combination of change detection and metachange detection algorithms are ϵ=0.7,w=100 (L=500), ϵ=0.7,w=200 (L=1000), and ϵ=0.7,w=400 (L=2000). Table 2 shows that MCD-S outperforms SMDL-MC overall because MCD-S deals with metachanges along state directly in terms of MCAS, whereas SMDL-MC only quantifies the difference in code lengths between situations where there is a change and where there is no change.

**Figure 8:**
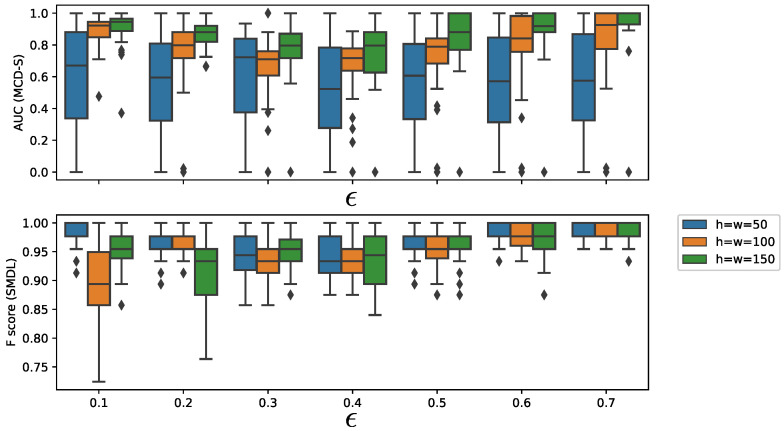
Dependency of AUC on threshold parameter ϵ for SMDL [8] and window size *h* of MCD-S on Synthetic Dataset 2.

We further investigated the effects of window size *h* and threshold parameters of the change detection algorithms. We chose SMDL [8] for change detection. Figure 8 shows the dependency of AUC on *h* and threshold parameter ϵ of SMDL. The interval length was set to L=500, threshold parameter was set to ϵ=0.1,0.2,0.3,0.4,0.5,0.6,0.7, and h=w=50,100,150, where *w* is the window parameter of SMDL. Figure 8 (top and bottom) shows the dependency of AUC of MCD-S on the threshold parameter ϵ of SMDL and the dependency of F-score of SMDL on ϵ, respectively. We observe in Figure 8 (top) that AUC of MCD-S decreases between ϵ=0.2 and 0.4, but, when ϵ exceeds 0.4, AUC begins to increase for h=50,100,150. This reflects the fact that there are many local maximum points of the change scores of SMDL, leading to false alarms of change points around ϵ=0.2–0.4. It is noticeable that F-scores of SMDL decrease for ϵ=0.1(h=100), and for ϵ=0.2(h=150), but AUCs of MCD-S do not do so much. This is because SMDL detects many false positive change points, but it detects the metachange point accurately.

As for the dependency of AUC on window size *h*, we observe that AUC generally increases as *h* increases for the same ϵ.

### 4.3. Synthetic Dataset 3 (Metachanges Along Time and State)

We generated a data stream that contained metachanges along both time and state. The stream consisted of two subsequences. The former part repeated changes of mean. Each instance was drawn from Equation (20) with $L=L_{1}$. We repeated the procedure for 50 times and obtained a subsequence with length $100L_{1}$. The latter part comprised the following four parts, each with length $L_{2}$:

$$y_{t}∼\left\{\begin{array}{cc} N(0,0.1) & (t=100L_{1}+1,\ldots ,100L_{1}+L_{2}),\\ N(0.45,0.1) & (t=100L_{1}+L_{2}+1,\ldots ,100L_{1}+4L_{2}). \end{array}\right.$$

In total, we obtained a data stream with length $100L_{1}+4L_{2}$. A metachange along both time and state occurred at $t=100L_{1}+L_{2}+1$. We chose lengths $L_{1}$ and $L_{2}$ among 400, 450, and 500.

We detected the metachange in the following three ways: we first detected change points with the same algorithms as in Section 4.2, and then detected the metachanges with MCD-T, MCD-S, and MCD. The parameters of the change detection algorithms were tuned as in Section 4.2. The ranges of parameters were the same as those in Section 4.2. except that, for SMDL, the threshold parameter ϵ=0.05,0.1,0.15 for all combinations of $L_{1}$ and $L_{2}$. The parameter of MCD-T was selected among r=0.1,0.2,0.3 and MCD-S was among $h=0.1L_{1},0.2L_{1}$. The window size of SMDL were selected among w=h, and the maximum tolerant delay was $\tau =L_{2}$. We chose the weight parameter λ in Equation (17) among λ=0.001,0.01,0.1,1,5,10. For VD, the buffer and reservoir sizes (*B* and *R*) were selected among 16,24,32. All the parameters were selected with grid search for the AUCs of metachange detection to be maximum.

Table 3 shows the average AUC values. Table 3a–c show average AUC values with MCD-T, MCD-S, and MCD. Table 3a shows that MCD combined with SMDL as the change detection algorithm outperforms MCD-S and MCD-T.

Table 4 shows the best parameters for each combination of intervals. We observe that the more intensive a metachange along time is, the bigger *r* is and the less λ becomes. These results reflect the fact that it is necessary to adapt to recent data, and MCAT increases in such a situation, leading to the decrease of λ.

### 4.4. Real Dataset: Human Action Recognition Data

**Figure 9:**
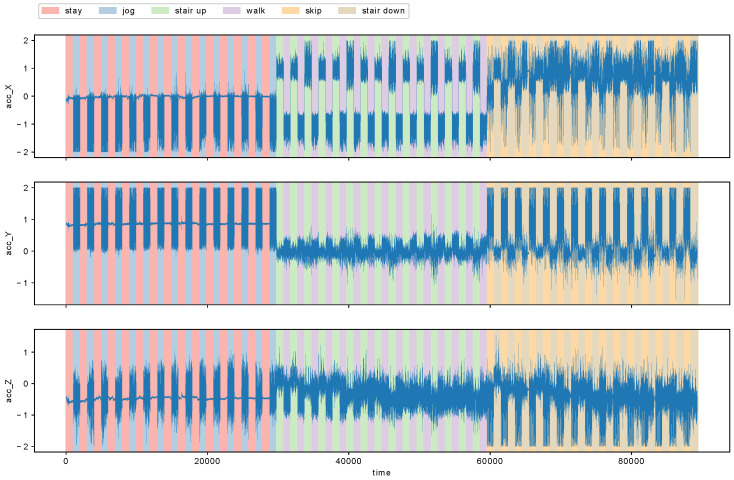
Human action recognition data for Person06023. Each row represents accelerations for *x*-, *y*-, and *z*-axes, respectively.

We applied MCD to the detection of metachanges in human action recognition data called HASC-PAC2016 dataset [28] (HASC-PAC2016 dataset is publicly available at http://hub.hasc.jp/). The data were collected from the Human Activity Sensing Consortium (HASC, http://hasc.jp/). HASC-PAC2016 dataset contains sequences of acceleration data for three axes, and each sequence is segmented into one of six action labels: “stay”, “walk”, “jog”, “skip”, “stair up”, (go upstairs) and “stair down” (go downstairs). For this experiment, we aimed to evaluate the effectiveness of our proposed algorithm MCD by using a data stream with ground truth of “changes of action changes” and “changes of intervals of actions”. The former corresponds to metachanges along state, and the latter to metachanges along time. We combined each action into a data stream as follows: first, we repeated “stay” and “walk” alternately for 15 times; then “jog” and “skip” for 15 times; and, finally, “stair up” and “stair down” for 15 times. We repeated each pair of actions for 15 times because “stair up” and “stair down” have only 15 files, which are the fewest in all the six actions. We obtained a data stream of length 89,324. Table 5 shows the files used for a participant named Person06023. We read the files sequentially in alphabetical order for each action. Figure 9 shows the data stream we obtained. Here, acc_X, acc_Y, and acc_Z represent accelerations for *x*-, *y*-, and *z*-axes, respectively.

**Figure 10:**
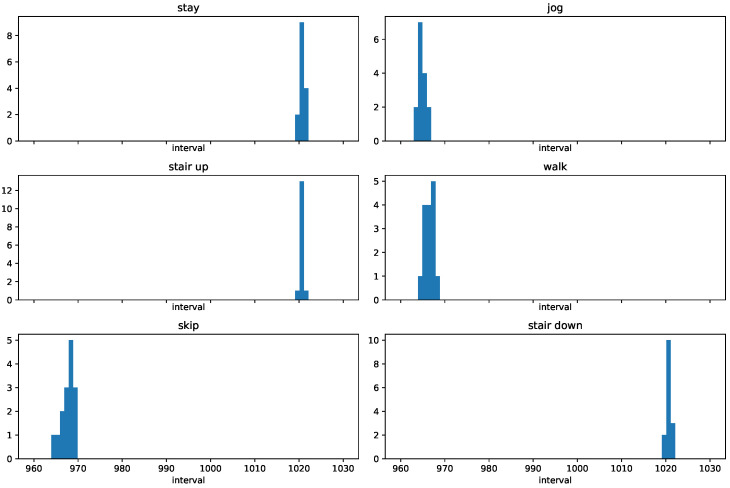
Histograms of intervals for each action label.

First, we detected change points with SMDL [8]. It was a challenge to determine the hyperparameters of SMDL—window size *w* and threshold parameter ϵ—in an online change detection. We tuned *w* and ϵ with the remaining dataset for Person06023, which alternated “stay” and “walk” four times, and “jog” and “skip” likewise. Although this dataset lacked “stair up” and “stair down”, we thought that it was enough to estimate the best configuration of *w* and ϵ. We calculated F-score as described in Section 4.2 for the change points between different action labels. We selected w=900 and ϵ=0.75 among w∈{500,600,700,800,900,1000} and ϵ∈{0,0.25,0.5,0.75,1}. Figure 10 shows histograms of intervals for each action label. We observe in Figure 10 that most of the intervals are around 960–970 for “jog”, “walk”, and “skip”, whereas, for “stay”, “stair up”, and “stair down”, the intervals are around 1020. We can see that w=900 was enough to detect changes.

We applied SMDL to the stream and obtained the estimated change scores $\left\{\Psi_{t}\right\}$ at each time point. We calculated $\Psi_{t}$ with the multivariate normal distribution. Specifically, $\Psi_{t}$ is calculated as

(21) $$\begin{array}{cc} \Psi_{t} & =\frac{1}{2}\log\frac{|{\hat{\Sigma }}_{0}{|}^{2}}{|{\hat{\Sigma }}_{1}\left|\right|{\hat{\Sigma }}_{2}|}+\frac{1}{2w}\log\frac{C_{2w}}{C_{w}^{2}}\\ \; & \;+\frac{1}{2w}\left\{\sum_{i=t-w}^{t+w}{\left(y_{i}-{\hat{\mu }}_{0}\right)}^{⊤}{\hat{\Sigma }}_{0}^{-1}\left(y_{i}-{\hat{\mu }}_{0}\right)\right.\\ \; & \;\;\left.-\sum_{i=t-w}^{t-1}{\left(y_{i}-{\hat{\mu }}_{1}\right)}^{⊤}{\hat{\Sigma }}_{1}^{-1}\left(y_{i}-{\hat{\mu }}_{1}\right)-\sum_{i=t}^{t+w}{\left(y_{i}-{\hat{\mu }}_{2}\right)}^{⊤}{\hat{\Sigma }}_{2}^{-1}\left(y_{i}-{\hat{\mu }}_{2}\right)\right\}, \end{array}$$

where ${\hat{\mu }}_{0}=1/\left(2w+1\right)\sum_{i=t-w}^{t+w}y_{i}$, ${\hat{\mu }}_{1}=1/w\sum_{i=t-w}^{t-1}y_{i}$, and ${\hat{\mu }}_{2}=1/\left(w+1\right)\sum_{i=t+1}^{t+w}y_{i}$. ${\hat{\Sigma }}_{0}=\frac{1}{2w}\sum_{i=t-w}^{t+w}\left(y_{i}-{\hat{\mu }}_{0}\right){\left(y_{i}-{\hat{\mu }}_{0}\right)}^{⊤}$, ${\hat{\Sigma }}_{1}=\frac{1}{w}\sum_{i=t-w}^{t-1}\left(y_{i}-{\hat{\mu }}_{1}\right){\left(y_{i}-{\hat{\mu }}_{1}\right)}^{⊤}$, and ${\hat{\Sigma }}_{1}=\frac{1}{w+1}\sum_{i=t}^{t+w}\left(y_{i}-{\hat{\mu }}_{2}\right){\left(y_{i}-{\hat{\mu }}_{2}\right)}^{⊤}$.

Note that $C_{w}$ in Equation (21) is the normalizer of the NML code length [29,30]:

(22) $$\begin{array}{cc} \log C_{w} & =-\left(m+1\right)\log\frac{m}{2}+\frac{m}{2}\log\mu_{\max}-\frac{m^{2}}{2}\log\sigma_{\min}+\frac{mw}{2}\log\frac{w}{2e}\\ \; & \;-\log\Gamma \left(\frac{m}{2}\right)-\log\Gamma_{m}\left(\frac{w-1}{2}\right), \end{array}$$

where *m* is the dimension of the data stream, Γ is the gamma function, and $\Gamma_{m}$ is calculated as

$$\Gamma_{m}\left(x\right)=\pi^{\frac{m(m-1)}{4}}\prod_{j=1}^{m}\Gamma \left(x+\frac{1-j}{2}\right).$$

We set $\mu_{\max}=50$ and $\sigma_{\min}=0.005$.

Next, we defined the ground truths for metachanges along state at two time points where the changes of action label changes occurred: t= 29,752 from “jog” to “stair up”, and t= 59,588 from “walk” to “skip”. Moreover, we also defined the ground truths for metachanges along time at time points where the changes of intervals occurred. We see in Figure 10 that the distributions are significantly different between four types of “changes of action changes”: from “stay” to “jog”, from “jog” to “stair up”, from “stair up” to “walk”, and from “skip” to “stair down”.

**Figure 11:**
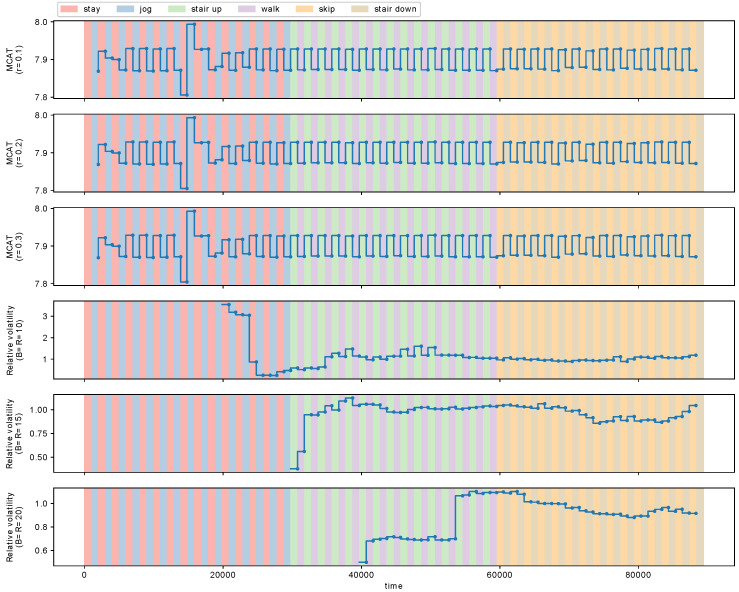
MCAT with MCD-T (r=0.1,0.2,0.3) and the relative volatility with the volatility detector [13] (B=R=10,15,20).

We detected metachanges along time with MCD-T and volatility detector (VD) [13], and compared them. Figure 11 shows the estimated MCAT with MCD-T and the relative volatility with VD. The parameter of MCD-T was set to r=0.1,0.2,0.3, whereas one of VD was B=R=10,15,20. Figure 11 shows the results.

We observe in Figure 11 that MCAT detects the metachanges along time between the four action pairs, respectively, for r=0.1,0.2, and 0.3. However, the relative volatility fails to detect some of these metachanges along time.

**Figure 12:**
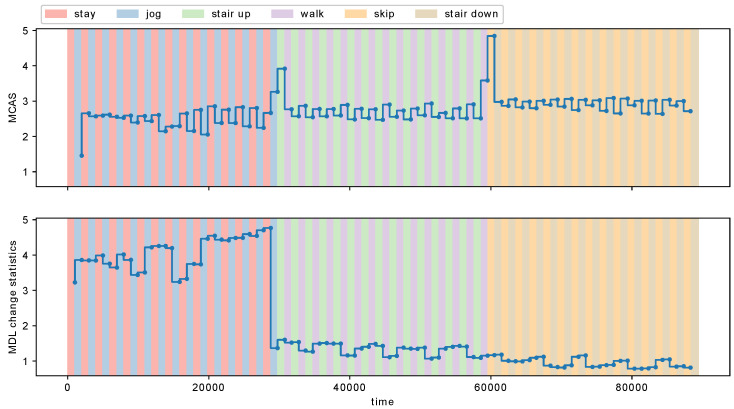
MCAS of MCD-S (h=900) and the MDL change statistics (w=900).

We detected metachanges along state with MCD-S and the change rate of the MDL change statistics [8]. Figure 12 shows the estimated MCAS with MCD-S and the MDL change statistics. We observe in Figure 12 that both MCD-S and the MDL change statistics detect a time point around t= 29,752 from “jog” to “stair up”. However, the MDL change statistics do not change significantly at a time point around t= 59,588, where a metachange along state happened from “walk” to “skip”. It indicates that the change rate of the MDL change statistics failed to detect the metachange along state around t= 59,588, whereas MCD-S detected it successfully.

In summary, the proposed algorithm MCD detected metachanges along both time and state more accurately than other methods.

### 4.5. Real Dataset: Production Condition Data

**Figure 13:**
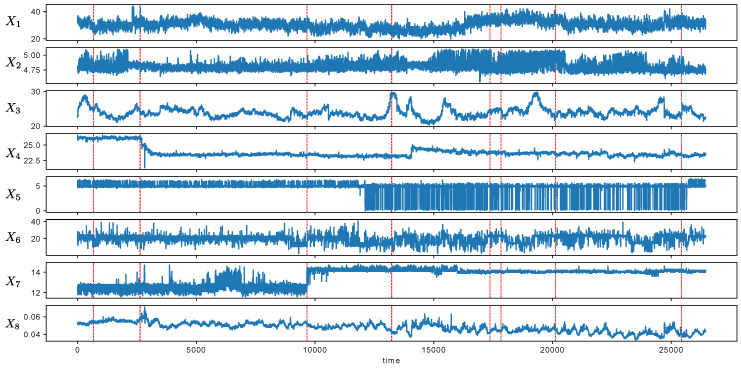
Data stream of the production condition data. Red dashed line indicates the time points where the important events occurred.

We applied MCD to the detection of metachanges in the production condition data. The data were collected from a factory of a manufacturing company. Each datum comprised eight attributes, and the length of the stream was 26,450. The factory reported that important events occurred 10 times during the study period, at t= 668, 2634, 2635, 9663, 13,230, 13,231, 17,372, 17,832, 20,131, and 25,441. Figure 13 shows the attributes from the stream. The dashed line indicates the time points where important events occurred. We investigated whether the detected metachanges were signs of important events, and we finally concluded that it might be true. The details are as follows.

Figure 13 shows that the scales of attributes were different. Hence, we normalized each attribute *X* to (X−μ)/σ, where μ and σ are the sample mean and standard deviation, respectively, which were calculated with the first 250 time points. First, we applied SMDL [8] to the stream and obtained the estimated change scores $\left\{\Psi_{t}\right\}$ at each time. We calculated $\Psi_{t}$ with the multivariate normal distribution in Equation (21). The window sizes *w* of SMDL and *h* of MCD were set to w=h=250 by field knowledge that it roughly represents a unit of production. Moreover, $\mu_{\max}$ and $\sigma_{\min}$ in Equation (22) were set to 60 and 0.001, respectively. Next, we detected change points $t_{1},\;t_{2},\;\ldots$ as time points where the change scores $\Psi_{t_{i}}$ were locally maximum within an interval where $\Psi_{t}>\epsilon$. We set ϵ=0.3 when the total change points detected was less than 0.5% of the total length. It is a business demand by a factory, and so there were not many alarms. The number of detected change points was 97 (0.37%). Finally, we determined the discounting parameter *r* and the weight parameter λ of MCD in Equation (17) with the first 5000 time points. We selected r=0.1 and λ=0.2 so that the AUC score at t=2634 and t=2635 would be the maximum. The AUC score was calculated using Equations (18) and (19).

**Figure 14:**
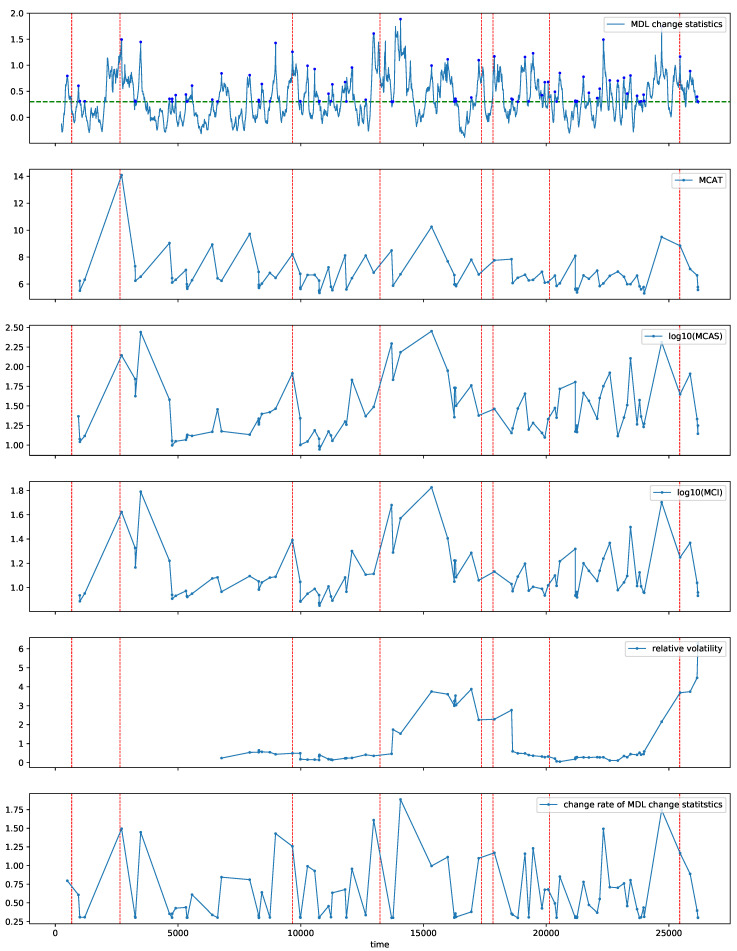
Metachange statistics of the production condition data: (**top**) the MDL change statistics $\left\{\Psi_{t_{i}}\right\}$. Blue dots show change points $\left\{t_{i}\right\}$, where $\Psi_{t_{i}}>\epsilon$; (**second**) estimated MCAT $a_{t_{i}}$; (**third**) estimated logarithm of MCAS $\log_{10}b_{t_{i}}$; (**fourth**) estimated logarithm of integrated metachange statistics (MCI) $\log_{10}s_{t_{i}}$; (**fifth**) relative volatility [13]; and (**bottom**) change rate of the MDL change statistics $|\left(\Psi_{t_{i}}-\Psi_{t_{i-1}}\right)/\Psi_{t_{i-1}}|$. h=w=250,ϵ=0.3,B=R=10,λ=0.2.

Figure 14 shows the MDL change statistics $\left\{\Psi_{t}\right\}$ calculated with SMDL [8] (Figure 14, top), the estimated MCAT $a_{t_{i}}$ (Figure 14, second), logarithm of the estimated MCAS $\log_{10}b_{t_{i}}$ (Figure 14, third), and logarithm of the estimated MCI $\log_{10}s_{t_{i}}$ (Figure 14, fourth). We also estimated the relative volatility with VD [13,25] (Figure 14, fifth) and the change rate of the MDL change statistics $|\left(\Psi_{t_{i}}-\Psi_{t_{i-1}}\right)/\Psi_{t_{i-1}}|$ (Figure 14, bottom) for comparison in detecting metachanges along both time and state. For VD, the buffer size *B* and the reservoir size *R* were both set to 10. In Figure 14 (top), the red points indicate the detected change points.

We summarize what can be seen for metachange statistics in Figure 14 as follows:

- t=9663: The trend of MCI increases roughly after t=5000, which can be interpreted as a combination of MCAT and MCAS in Figure 14. The relative volatility and the change rate of the MDL change statistics do not show such a significant sign.
- t= 13,230, 13,231, 17,372, 17,832: For time points between t= 10,000 and t= 15,000, the trend of MCI increases. It is also due to the combination of MCAT and MCAS, but is more influenced by MCAS. It might also be a sign of important events at t= 17,372 and 17,832 as well as t= 13,230 and t= 13,231. The relative volatility increases after t= 13,231, which might be a sign of the important event at t= 17,372. However, the change rate of the MDL change statistics does not show such a significant sign.
- t= 25,440: For time points between t= 20,000 and t= 25,000, the trend of MCI increases with large fluctuations. It is also more influenced by MCAS. It might also be a sign of important events at t= 25,440. The relative volatility increases for the time points, but the change rate of the MDL change statistics does not show such a significant sign.

In summary, we can observe a sign of metachange for each important event. We therefore infer that there might have been some symptoms that should be analyzed using field knowledge.

## 5. Conclusions

We propose the concept of *metachanges* along time and state in data streams, and we introduce *metachange statistics* to quantify metachanges from a unified view with MDL. The key idea of our proposed method is to encode the time intervals and change of states with code lengths in the same fashion. Next, we introduce the novel methodology of MCD. Using synthetic datasets, we empirically demonstrated that the proposed algorithm was highly effective in detecting metachanges along time and state. Using a real dataset, we demonstrated that the proposed algorithm could detect metachanges in both time and state, some of which were overlooked by VD [13] and the MDL change statistics [8]. The estimated metachange statistics might have been a sign of important events.

Future work will be directed toward the theoretical guarantee of metachange statistics, especially integrated metachange statistics. We will also consider how to adapt to a non-stationary data stream by updating the weight parameter λ in Equation (17). Other research directions might lie in the extension of metachange statistics to transient periods between change points. Furthermore, metachange detection of model structure change and its change sign is another interesting line of research. 

## Figures and Tables

**Table 1:** Average area under the curve (AUC) scores on Synthetic Dataset 1 ($r=0.2,\tau =5L_{2}$). Boldfaces describe best performances.

$L_{1}$	$L_{2}$	SEED		SMDL	
		MCD-T	VD	MCD-T	VD
100,000	50,000	0.603±0.180	0.458±0.199	0.500±0.060	0.313±0.160
100,000	10,000	0.621±0.147	0.310±0.167	0.710±0.254	0.463±0.113
100,000	5000	0.645±0.129	0.328±0.152	0.668±0.223	0.416±0.164
100,000	1000	0.651±0.110	0.275±0.135	0.512±0.123	0.448±0.107
100,000	500	0.697±0.140	0.336±0.140	0.660±0.111	0.506±0.138
50,000	100,000	0.788±0.093	0.647±0.107	0.729±0.067	0.639±0.107
50,000	10,000	0.671±0.103	0.280±0.130	0.605±0.171	0.556±0.060
50,000	5000	0.708±0.087	0.293±0.144	0.617±0.183	0.546±0.146
50,000	1000	0.718±0.067	0.294±0.140	0.655±0.161	0.501±0.144
50,000	500	0.767±0.110	0.316±0.133	0.686±0.074	0.470±0.157
10,000	100,000	0.863±0.059	0.794±0.058	0.877±0.068	0.791±0.015
10,000	50,000	0.834±0.050	0.735±0.050	0.876±0.066	0.823±0.026
10,000	5000	0.723±0.040	0.344±0.250	0.658±0.159	0.498±0.084
10,000	1000	0.781±0.014	0.375±0.260	0.689±0.083	0.444±0.077
10,000	500	0.809±0.063	0.391±0.256	0.671±0.163	0.520±0.070
5000	100,000	0.856±0.060	0.796±0.067	0.854±0.071	0.798±0.036
5000	50,000	0.825±0.047	0.726±0.062	0.875±0.032	0.708±0.043
5000	10,000	0.777±0.030	0.575±0.139	0.716±0.060	0.630±0.031
5000	1000	0.783±0.009	0.436±0.257	0.709±0.009	0.353±0.098
5000	500	0.816±0.054	0.493±0.269	0.839±0.191	0.413±0.097
1000	100,000	0.872±0.072	0.814±0.072	0.836±0.036	0.812±0.036
1000	50,000	0.844±0.059	0.754±0.061	0.947±0.037	0.810±0.027
1000	10,000	0.802±0.022	0.668±0.050	0.873±0.049	0.805±0.023
1000	5000	0.801±0.014	0.648±0.064	0.895±0.053	0.812±0.053
1000	500	0.816±0.048	0.560±0.242	0.711±0.141	0.409±0.108
500	100,000	0.876±0.068	0.831±0.063	0.830±0.079	0.820±0.023
500	50,000	0.845±0.062	0.767±0.062	0.836±0.044	0.818±0.010
500	10,000	0.827±0.051	0.676±0.047	0.872±0.023	0.822±0.016
500	5000	0.830±0.047	0.663±0.042	0.864±0.047	0.819±0.017
500	1000	0.830±0.050	0.612±0.100	0.935±0.022	0.853±0.095

**Table 2:** Average AUC scores on Synthetic Dataset 2. The first and second headers represent change detection and metachange detection algorithms, respectively. Boldfaces describe best performances.

*L*	SMDL		CF		BOCPD		ADWIN2	
	MCD-S	SMDL-MC	MCD-S	SMDL-MC	MCD-S	SMDL-MC	MCD-S	SMDL-MC
500	0.887±0.100	0.795±0.156	0.874±0.111	0.851±0.170	0.701±0.318	0.572±0.332	0.797±0.186	0.853±0.114
1000	0.921±0.018	0.905±0.012	0.912±0.042	0.830±0.052	0.751±0.323	0.743±0.291	0.834±0.094	0.847±0.048
2000	0.970±0.010	0.953±0.011	0.912±0.033	0.843±0.022	0.829±0.124	0.821±0.138	0.951±0.032	0.887±0.046

**Table:** (**a**) Metachange detection along time.

$L_{1}$	$L_{2}$	SMDL		CF		BOCPD		ADWIN2	
		MCD-T	VD	MCD-T	VD	MCD-T	VD	MCD-T	VD
400	450	0.867±0.022	0.845±0.013	0.818±0.031	0.815±0.025	0.843±0.053	0.825±0.038	0.839±0.048	0.806±0.043
400	500	0.871±0.021	0.867±0.021	0.815±0.040	0.812±0.041	0.831±0.049	0.814±0.033	0.823±0.048	0.826±0.038
450	400	0.813±0.024	0.804±0.017	0.795±0.044	0.784±0.029	0.810±0.038	0.805±0.031	0.805±0.052	0.812±0.035
450	500	0.872±0.014	0.863±0.019	0.822±0.039	0.819±0.047	0.847±0.032	0.829±0.042	0.816±0.044	0.815±0.034
500	400	0.874±0.024	0.867±0.024	0.837±0.016	0.813±0.045	0.822±0.019	0.797±0.029	0.815±0.011	0.802±0.031
500	450	0.893±0.015	0.873±0.019	0.829±0.013	0.859±0.039	0.833±0.011	0.823±0.049	0.819±0.021	0.875±0.031

**Table:** (**b**) Metachange detection along state.

$L_{1}$	$L_{2}$	SMDL		CF		BOCPD		ADWIN2	
		MCD-S	SMDC-MC	MCD-S	SMDC-MC	MCD-S	SMDC-MC	MCD-S	SMDC-MC
400	450	0.901±0.012	0.857±0.014	0.823±0.013	0.833±0.024	0.855±0.021	0.858±0.031	0.809±0.015	0.867±0.011
400	500	0.923±0.016	0.911±0.023	0.813±0.011	0.812±0.014	0.852±0.034	0.851±0.028	0.805±0.036	0.798±0.024
450	400	0.895±0.022	0.875±0.011	0.835±0.021	0.809±0.033	0.855±0.034	0.853±0.025	0.809±0.033	0.892±0.031
450	500	0.917±0.017	0.905±0.023	0.842±0.039	0.825±0.047	0.837±0.051	0.819±0.042	0.838±0.044	0.615±0.034
500	400	0.875±0.024	0.863±0.022	0.822±0.032	0.813±0.045	0.810±0.026	0.797±0.022	0.729±0.024	0.702±0.023
500	450	0.865±0.021	0.823±0.028	0.715±0.038	0.723±0.049	0.728±0.045	0.706±0.038	0.694±0.042	0.675±0.031

**Table:** (**c**) Metachange detection along both time and state.

$L_{1}$	$L_{2}$	SMDL	CF	BOCPD	ADWIN2
		MCD	MCD	MCD	MCD
400	450	0.985±0.011	0.971±0.023	0.968±0.033	0.967±0.029
400	500	0.989±0.007	0.975±0.016	0.971±0.005	0.969±0.031
450	400	0.983±0.016	0.981±0.013	0.968±0.035	0.966±0.014
450	500	0.987±0.010	0.982±0.014	0.975±0.025	0.970±0.029
500	400	0.979±0.015	0.973±0.011	0.969±0.012	0.964±0.013
500	450	0.975±0.012	0.969±0.010	0.967±0.018	0.954±0.021

**Table 4:** Best parameters for each combination of intervals.

$L_{1}$	$L_{2}$	*r*	*w*	*h*	λ
400	450	0.2	$0.2L_{1}$	$0.2L_{1}$	0.1
400	500	0.3	$0.2L_{1}$	$0.2L_{1}$	0.01
450	400	0.1	$0.2L_{1}$	$0.2L_{1}$	0.1
450	500	0.2	$0.2L_{1}$	$0.2L_{1}$	0.1
500	400	0.3	$0.2L_{1}$	$0.2L_{1}$	0.01
500	450	0.1	$0.2L_{1}$	$0.2L_{1}$	0.1

**Table 5:** Files for generating a sequence of Person06023.

Action Label	Files
stay	HASC *N*-acc.csv (*N* = 0605581–0605595)
walk	HASC *N*-acc.csv (*N* = 0608420–0608434)
jog	HASC *N*-acc.csv (*N* = 0611173–0611187)
skip	HASC *N*-acc.csv (*N* = 0613411–0613425)
stair up	HASC *N*-acc.csv (*N* = 0615620–0615634)
stair down	HASC *N*-acc.csv (*N* = 0614162–0614166)
